# Supplementary material for: A Rapid, Simple, Trace, Cost-Effective, and High-Throughput Stable Isotope-Dilution Liquid Chromatography–Tandem Mass Spectrometry Method for Serum Methylmalonic Acid Quantification and Its Clinical Applications
Source: Diagnostics (Basel). 2022 Sep 20;12(10):2273. doi: 10.3390/diagnostics12102273 (PMC9600096; doi:10.3390/diagnostics12102273)
Supplement: Supplementary file 1 [file diagnostics-12-02273-s001.zip › diagnostics-1893560-supplementary.pdf]

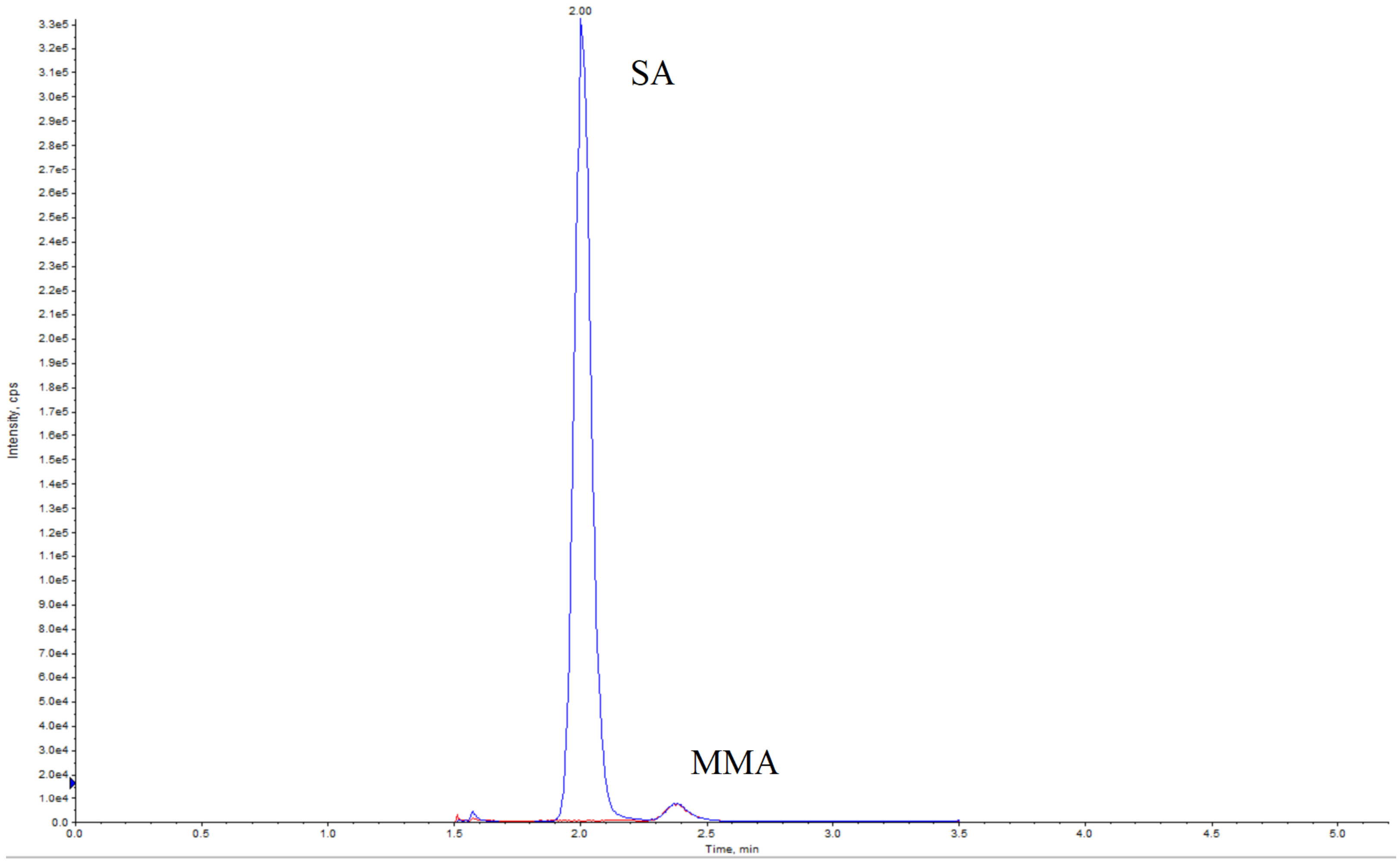

Figure S1: Chromatographic separation by isocratic elution with 95% A phase (0.1% formic acid water solution) and 5% B phase (methanol). This procedure needs drying and residue reconstitution by 5% methanol.
